# Comparative measurements of bone mineral density and bone contrast values in canine femora using dual-energy X-ray absorptiometry and conventional digital radiography

**DOI:** 10.1186/s12917-017-1047-y

**Published:** 2017-05-11

**Authors:** K. Lucas, I. Nolte, V. Galindo-Zamora, M. Lerch, C. Stukenborg-Colsman, B. A. Behrens, A. Bouguecha, S. Betancur, A. Almohallami, P. Wefstaedt

**Affiliations:** 1 Small Animal Hospital, University of Veterinary Medicine Hannover, Foundation, Bünteweg 9, D-30559 Hannover, Germany; 2 Small Animal Clinic, Faculty of Veterinary Medicine, National University of Colombia, Bogotá, Colombia; 3 Department of Orthopaedic Surgery, Hannover Medical School, Hanover, Germany; 4 Institute of Forming Technology and Machines, Leibniz University Hannover, Hannover, Germany

**Keywords:** Bone mineral density, DEXA, Digital radiography, Canine, Femur, Bone remodelling

## Abstract

**Background:**

Aseptic loosening due to bone remodelling processes after total hip replacement is one common cause for revision surgery. In human medicine, dual-energy X-ray absorptiometry (DEXA) is the gold standard for quantitative evaluation of bone mineral density, whereas in veterinary medicine conventional radiography is used for follow-up studies. Recently, a method has been described using digital X-ray images for quantitative assessment of grey scale values of bone contrast. Therefore, the aim of the present study was to evaluate the correlation of bone mineral density (BMD) measured by DEXA with grey scale values (GV) measured in digital X-ray images (RX50, RX66) ex vivo.

**Results:**

The measured GV in the chosen X-ray settings showed on average a good correlation (r = 0.61) to the measured BMD with DEXA. Correlation between the two X-ray settings was very good (r = 0.81). For comparisons among regions of interests (ROIs) a difference of 8.2% was found to be statistically significant, whereas in the case of RX50 and RX66 differences of 5.3% and 4.1% were found to be statistically significant.

**Conclusions:**

Results indicate that measuring absolute changes in bone mineral density might be possible using digital radiography. Not all significant differences between ROIs detectable with DEXA can be displayed in the X-ray images because of the lower sensitivity of the radiographs. However, direct comparison of grey scale values of the periprosthetic femur in one individual patient during the follow-up period, in order to predict bone remodelling processes, should be possible, but with a lesser sensitivity than with DEXA. It is important that the same X-ray settings are chosen for each patient for follow-up studies.

**Electronic supplementary material:**

The online version of this article (doi:10.1186/s12917-017-1047-y) contains supplementary material, which is available to authorized users.

## Background

In humans and dogs, severe damage of the hip joint is usually treated with total hip replacement (THR) [1]. Different prosthetic devices exist on the market. These include cemented, cementless and hybrid implants [2–8]. Complications following THR are luxation, infection, aseptic or septic loosening, femoral fracture and sciatic neurapraxia [9, 10]. Aseptic loosening is one common cause for revision surgery [10, 11]. Different reasons for aseptic loosening processes are assumed, including particle disease, micromotion and stress shielding [12]. Stress shielding is due to different load transfer because of the higher modulus (E) of the prosthesis compared to bone which leads to loss of bone mineral density. It is of utmost importance to diagnose loosening processes in good time in order to minimise patients’ pain and distress. Therefore, it is necessary to detect as soon as possible the amount and localisation of bone loss around the prosthesis, which is reflected in changes in bone mineral density [13].

According to the World Health Organisation (WHO), dual-energy X-ray absorptiometry (DEXA) is the gold standard for quantitative measurements of bone mineral density in human medicine [14, 15]. DEXA measurements are performed in the spine and in the hip for diagnosing osteoporosis [16]. To measure bone mineral density in the periprosthetic femur, the proximal part of the femur surrounding the prosthesis is usually divided into 7 regions of interest called Gruen zones in human medicine, according to the description of Gruen et al.[17].

However, DEXA devices are not commonly available in veterinary medicine and are therefore rarely used for follow-up studies after THR in dogs. Conventional radiography is usually applied in veterinary medicine to predict the clinical outcome. Usually, X-ray images are used to evaluate parameters such as femoral cortical thickening/atrophy, signs of fissure/fracture, radiolucent lines, thickness of the cement mantle, position of the implant parts and subluxation [4, 7, 10, 18]. To the authors’ knowledge, the question if bone remodelling processes around femoral implants are reliably detected with digital radiography in dogs has not been answered yet. One study examined these processes after THR in dogs using X-ray images from digital radiography for evaluating grey scale values (GV) [19]. For evaluating bone remodelling processes in the canine femur, Gruen zones were adapted and reduced to five region of interests (ROIs) [19, 20]. Another study reported a correlation of mean grey value in digitalised and digital images of conventional and digital radiography of bovine and equine bone specimens with BMD assessed with DEXA of 0.910 and 0.937, respectively [21]. These results indicate that also differences in bone mineral density as found in the different regions of the canine femur should be detectable with digital radiography. To the authors’ knowledge, no comparable study exists in dogs. Therefore, the aim of this study was to compare BMD from DEXA measurements with GV in digital radiography and to evaluate the influence of different X-ray settings on the mean GV in canine carcass femora.

## Methods

### Material

Bones were obtained from cadavers of 15 dogs (mean body weight = 26.4 kg; SD = 5.6 kg; 3 Alsatians, 3 mixed breeds, 2 Golden Retrievers, 2 Small Muensterlaenders, 2 Irish Setters, 2 English Bulldogs, 1 Border Collie) that were euthanised due to medical reasons not related to this study and ceded for use for research purposes either to the Institute of Anatomy or the Small Animal Hospital of the University of Veterinary Medicine Hannover, Foundation. Written owner consent was obtained by the Small Animal Hospital of the University of Veterinary Medicine Hannover, Foundation. Both explanted femora from all 15 dogs were measured using DEXA and conventional X-ray for quantitative analysis of bone mineral density (DEXA) and grey scale values (X-ray) as well. Up to the time of examination, the femora were wrapped in cloth, soaked with 0.9% saline solution and frozen at −20 °C.

### DEXA measurements

**Fig. 1:**
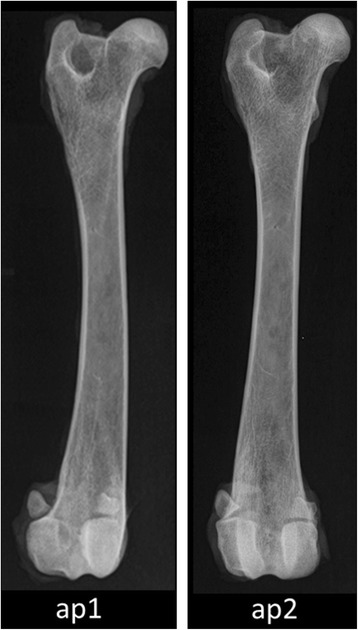
Anterior-posterior positions ap1 and ap2. X-ray of patient no. 8 (Alsatian, 24 kg) right femur in anterior-posterior positions (ap1, ap2). ap1: anterior-posterior position, femur rotated medially about 20°; ap2: conventional anterior-posterior position like for HD diagnosis

DEXA measurements were performed with the scanning mode “metal hip removal” of a Hologic Discovery A S/N 80600 device (Hologic Inc., Waltham, MA, USA). The afore-mentioned mode was chosen to create data that are comparable with studies investigating patients after total hip replacement although the bones in this study did not include a metal implant. Two different anterior-posterior positions were chosen to evaluate whether there is an influence of different rotations of the respective femur. For the anterior-posterior positioning 1 (Fig. 1, ap1), the femur was rotated medially about 20° in order to prevent the femoral neck from superimposing with the femoral head. This is the standard position for human patients. The conventional anterior-posterior positioning (Fig. 1, ap2) is commonly used for evaluating hip dysplasia. Additionally, two measurements were carried out in mediolateral projection (ml1 and ml2) with the femur laterally rotated 90° to ap1 and ap2, respectively (see online Additional file 1: ml1_ml2.tif). Evaluation of DEXA images was performed using the integrated software of the afore-mentioned DEXA device (Hologic Inc., Waltham, MA, USA).

### X-ray examination

For digital X-ray examination (S/N 09000024; Philips Medical Systems DMC GmbH; Hamburg; Germany), the femora were positioned the same way as for the DEXA measurements. Two different X-ray settings (RX50 = 50 kV/6.3 mAs and RX66 = 66 kV/8 mAs) commonly used for X-ray imaging of the hind limb in living dogs were applied. After data export as *.jpeg, radiographs were analysed using the freely available image processing software ImageJ (Rasband, W.S., ImageJ, U. S. National Institutes of Health, Bethesda, Maryland, USA, http://imagej.nih.gov/ij/).

### Zonal classification

**Fig. 2:**
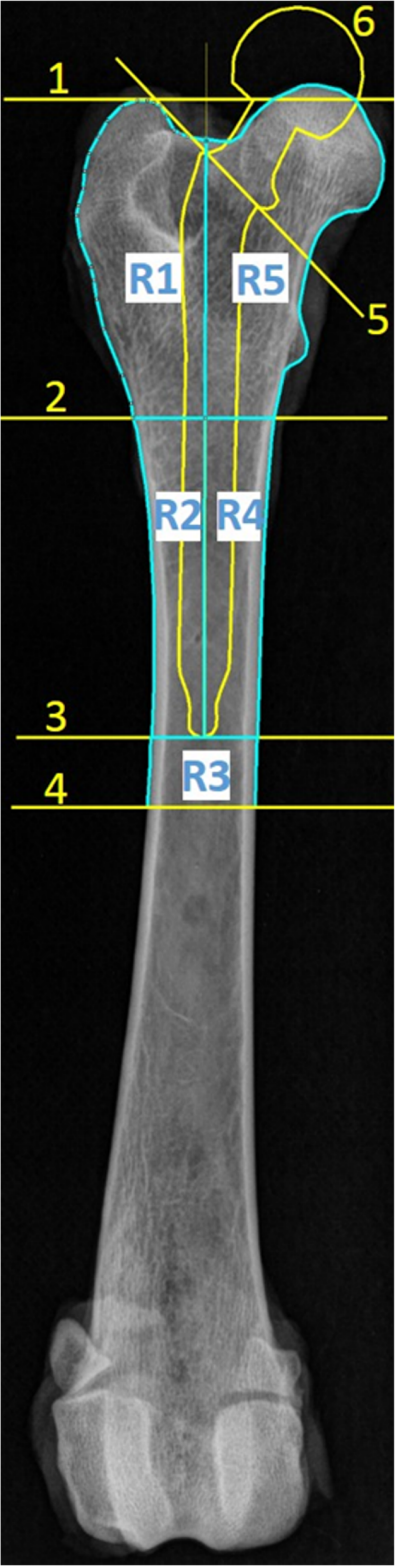
Definition of ROIs. X-ray image (66 kV, 8mAs) in anterior-posterior position ap2, right femur of patient no. 8 (Alsatian, 24 kg) as an example. R1 – R5 = Region of Interest 1–5; 1: greater trochanter line, 2: bisector line, 3: tip of prosthesis, 4: 1 cm distal tip of prosthesis, 5: femoral head dissection line, 6: template of a prosthesis

**Fig. 3:**
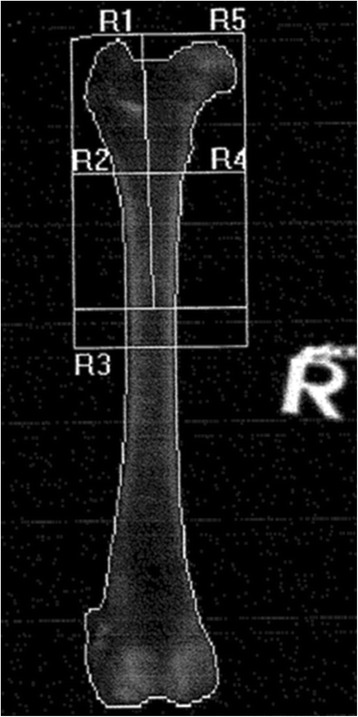
DEXA scan ap2. DEXA scan in anterior-posterior position ap2 (Hologic Discovery A S/N 80600), right femur of patient no 8 (Alsatian, 24 kg) as an example. R1 – R5 = Region of Interest 1–5

For analyses of DEXA images as well as of X-ray images, Gruen Zones [17] were adapted to the canine femur and reduced to 5 regions of interest (ROIs) as described previously (Figs. 2 and 3) [19, 20]. For defining ROI sizes within X-ray data sets by means of ImageJ, a virtual prosthesis template of appropriate size was generated and positioned as an overlay on the respective femur image (Fig. 2). The axis of the virtual prosthesis was aligned to the femoral axis. Approximately 2–3 mm space for a cement mantle was left free between the prosthesis and the inner border of the compacta. The prosthesis was inserted into the femur until the neck closed up with a line where the femoral head was virtually dissected (Fig. 2; 5). A horizontal line perpendicular to the femur axis was inserted to mark the tip of the virtual prosthesis (Fig. 2; 3). A bisector line (Fig. 2; 2) was drawn in the middle between the tip of the prosthesis and a second line positioned at the proximal margin of the greater trochanter (Fig. 2; 1). By halving the shaft lengthwise, ROI1 and ROI2 (lateral) as well as ROI4 and ROI5 (medial) were generated in the proximal femur. ROI3, with a fixed height of 1 cm (distance between line 3 and line 4, Fig. 2), was located directly distal of the tip of the virtual prosthesis. These 5 ROIs were determined separately for every femur. The location and dimension of every ROI was exactly measured and also applied for DEXA analysis to ensure the evaluation of the same area in the two different modalities.

### Statistical analysis

Statistical analysis was performed with SPSS® (SPSS Inc. Chicago, IL, USA). Pearson’s correlation coefficient between DEXA and RX50, between DEXA and RX66 and between RX50 and RX66 was calculated for each position and every ROI. Correlation was considered as follows: r = 0 → no correlation; 0 < r ≤ 0.2 → very poor; 0.2 < r ≤ 0.4 → poor; 0.4 < r ≤ 0.6 → moderate; 0.6 < r ≤ 0.8 → good; 0.8 < r < 1 → very good; r = 1 perfect. One-way ANOVA followed by a Tukey post hoc test was performed to analyse differences between the different ROIs for DEXA, RX50 and RX66 and for each positioning.

## Results

Measured bone mineral density (DEXA) and grey scale values (X-ray) for all ROIs in both ap1 and ap2 are displayed in Tables 1 and 2 (mean value (M) ± standard deviation (SD), coefficient of variation [CV]). Results of the measurements in mediolateral positioning ml1 and ml2 are presented in the online supplements (Additional file 2: BMD_GV_ml1_ml2.docx, Additional file 3: Boxplots_ml1.tif, Additional file 4: Boxplots_ml2.tif, Additional file 1: ml1_ml2.tif).

**Table 1:** Measured bone mineral density (DEXA) and grey scale values (X-ray) for the regions of interest 1–5 (ROI1 – ROI5) in ap1: mean value (M) ± standard deviation (SD), coefficient of variation [CV]

	Anterior-posterior position ap1		
Region of Interest	DEXA (g/cm^2^)	RX50 (grey scale value)	RX66 (grey scale value)
ROI1	0.62 ± 0.04 [0.07]	123.28 ± 9.65 [0.08]	118.78 ± 7.25 [0.06]
ROI2	0.74 ± 0.06 [0.08]	127.84 ± 7.07 [0.05]	122.24 ± 4.93 [0.04]
ROI3	0.79 ± 0.10 [0.13]	125.64 ± 7.51 [0.06]	119.09 ± 6.64 [0.05]
ROI4	0.79 ± 0.07 [0.09]	128.58 ± 6.53 [0.05]	122.49 ± 4.97 [0.04]
ROI5	0.73 ± 0.05 [0.07]	129.81 ± 9.97 [0.08]	125.23 ± 7.78 [0.06]

**Table 2:** Measured bone mineral density (DEXA) and grey scale values (X-ray) for the regions of interest 1–5 (ROI1 – ROI5) in ap2: mean value (M) ± standard deviation (SD), coefficient of variation [CV]

	Anterior-posterior position ap2		
Region of Interest	DEXA (g/cm^2^)	RX50 (grey scale value)	RX66 (grey scale value)
ROI1	0.63 ± 0.04 [0.07]	122.73 ± 8.37 [0.07]	118.56 ± 7.11 [0.06]
ROI2	0.80 ± 0.07 [0.09]	132.10 ± 6.34 [0.05]	127.61 ± 5.21 [0.04]
ROI3	0.82 ± 0.11 [0.13]	127.39 ± 7.37 [0.06]	122.56 ± 7.42 [0.06]
ROI4	0.80 ± 0.07 [0.09]	129.42 ± 6.55 [0.05]	124.68 ± 5.99 [0.05]
ROI5	0.72 ± 0.06 [0.08]	129.20 ± 7.66 [0.06]	125.16 ± 6.43 [0.05]

### Correlation between DEXA and X-ray

For ap1, correlation between DEXA and RX50 was good in ROI3 (r = 0.8) and in ROI1 (r = 0.6), moderate in ROI4 (r = 0.5) and ROI5 (r = 0.54) and poor in ROI2 (r = 0.34). Correlation between DEXA and RX66 in ap1 was good in ROI3 (r = 0.72), moderate in ROI1 (r = 0.49), ROI4 (r = 0.49) and ROI5 (r = 0.47) and poor in ROI2 (r = 0.34).

For ap2, correlation between DEXA and RX50 was good in ROI3 (r = 0.8) and in ROI4 (r = 0.62) and moderate in ROI1 (r = 0.56), ROI2 (r = 0.58) and ROI5 (r = 0.57). Correlation between DEXA and RX66 for ap2 showed slightly better results than ap1, being good in ROI1 (r = 0.61), ROI2 (r = 0.66), ROI3 (r = 0.79) and ROI4 (r = 0.69) and moderate in ROI5 (r = 0.58). All correlations were highly significant (*p* < 0.01). Only in ap1 ROI2, where the correlation was poor for both DEXA – RX50 and RX66, respectively, were the results not significant (p > 0.05).

### Correlation between the different X-ray settings

Correlation between RX50 and RX66 was very good in ap1 ROI1 (r = 0.83) and ROI5 (r = 0.86), in ap2 ROI1 (r = 0.85) and ROI5 (r = 0.88). Correlation between RX50 and RX66 was good in ap1 ROI2 (r = 0.78), ROI3 (r = 0.66) and ROI4 (r = 0.63), in ap2 ROI2 (r = 0.78), ROI3 (r = 0.79) and ROI4 (r = 0.79). All correlations were highly significant (*p* < 0.01).

### Differences between ROIs

**Fig. 4:**
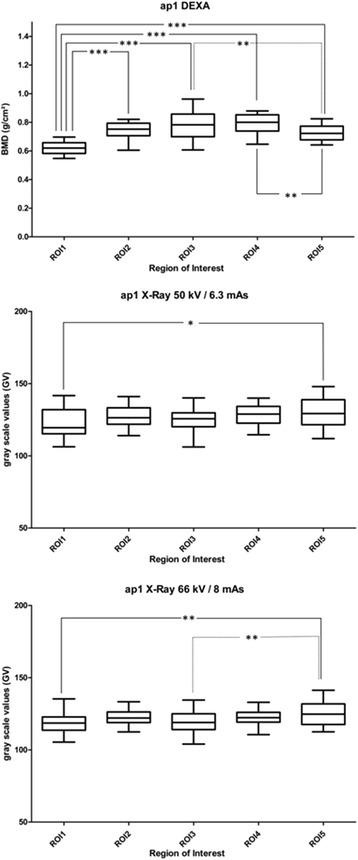
Box plots BMD and GV in ap1. Box plots (min to max, mean) for measured bone mineral density (BMD) in DEXA, and grey scale values (GV) in RX50 (X-ray 50 kV/6.3 mAs) and RX66 (X-ray 66 kV/8 mAs) for ROI1 – ROI5. One-Way ANOVA, * - *** statistically significant (*p* < 0.05 – *p* < 0.001)

**Fig. 5:**
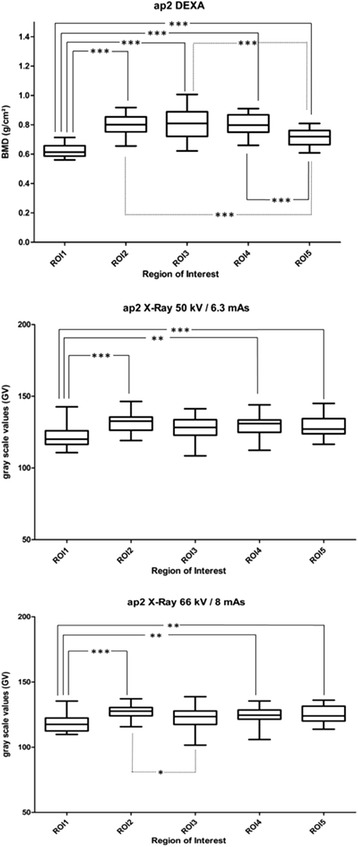
Box plots BMD and GV in ap2. Box plots (min to max, mean) for measured bone mineral density (BMD) in DEXA, and grey scale values (GV) in RX50 (X-ray 50 kV/6.3 mAs) and RX66 (X-ray 66 kV/8 mAs) for ROI1 – ROI5. One-Way ANOVA, * - *** statistically significant (*p* < 0.05 – *p* < 0.001)

Statistically significant differences are shown in Figs. 4 and 5 (* - *** = *p* < 0.05 – *p* < 0.001).

In ap1 statistically significant differences could be detected in the case of DEXA measurements for ROI1 compared to all other ROIs, whereas in RX50 and RX66 this was only the case between ROI1 and ROI5. The significance of differences when comparing ROI3 to ROI5 and ROI4 to ROI5 as seen in ap1 in DEXA could not be detected in RX50. RX66 detected differences between ROI3 and ROI5 as seen with DEXA, but not between ROI4 and ROI5.

For ap2, DEXA detected significant differences between ROI1 and all other ROIs and between all ROIs except for ROI1 compared to ROI5. Values measured in ROI2, ROI3 and ROI4 did not differ significantly. Similar to ap1, also in ap2 RX66 detected more significant differences than RX50. For ap2, differences in RX50 could be observed between ROI1 compared to ROI2, ROI1 compared to ROI4 and ROI1 compared to ROI5. The same holds true for RX66 ap2, and additionally differences between ROI2 compared to ROI3 were observed in RX66 ap2.

## Discussion

Both modalities used in this study (digital radiography and DEXA) are based on X-radiation and produce a 2-dimensional image of a 3-dimensional object. The produced image is the result of the radiation absorption of the respective tissue which depends on its density and thickness [22]. The higher the density and thickness of the tissue, the greater is the absorption. Depending on how much radiation reaches the detector, every pixel of the image is assigned a grey value depending on the attenuation value [22]. The difference between conventional radiography and DEXA is that the latter uses two different X-ray voltages from different sources at the same time (100 kV and 140 kV in this study). In contrast, conventional radiography only uses one source of energy (in this study 50 kV for RX50 or otherwise 66 kV for RX66). Thus, in DEXA every pixel includes two different attenuation values. This information is automatically converted to Bone Mineral Density (BMD) by the DEXA software (in g/cm^2^).

In the literature, the influence of the rotation of DEXA results is reported from −10.5% to + 2.8% and in individual cases up to 60% [23]. Although the positioning of the femora for the X-ray and DEXA examinations was performed with utmost care, it might be possible that the degree of rotation of the femur was not 100% the same between DEXA and X-ray due to the manual positioning. A positioning guide should be used in further studies to reduce bias particularly when examining living patients where correct positioning is more challenging due to the soft tissue covering the bone. Another methodical limitation of the study was that the definition of ROI geometry and position was based on the X-ray examinations. This setup was transferred to the DEXA images which could have led to small differences in the examined areas. For ap1 and ap2, both X-ray settings detected fewer significant differences between ROIs than DEXA. In veterinary medicine, the anterior-posterior position ap1, which is equivalent to the hip dysplasia examination, is the most common position used for follow-up studies. Variance of DEXA measurements were slightly higher (CV = 0.07–0.13) than in RX50 (CV = 0.05–0.08) or RX66 (CV = 0.04–0.08). The minimal detectable difference of BMD with DEXA amounted to 8.2% (ap1: ROI3 – ROI5 and ROI4 – ROI5). For RX50, the minimal difference was 5.3% (ap1: ROI1 – ROI5), whereas RX66 was able to detect changes in grey values with a difference of 4.1% (ap2: ROI2 – ROI3). In follow-up studies using DEXA after THR, statistically significant differences in BMD between ROIs after 6 months, 12 months and 2 years to baseline value 1 week postoperatively were detected between −11.54 and +10.6% [13, 24]. The only previous study evaluating grey scale values in follow-up radiography after THR in dogs detected a statistical significant difference at 10.74% 4 months after surgery [19]. Our results therefore show a clinical relevance for follow-up studies aiming to investigate changes in grey scale values of bone X-rays over time. However, this technique is of course less sensitive than DEXA. The chosen X-ray setting has an influence on the grey scale values. Lower X-ray current and voltage revealed slightly higher grey scale values in the same ROIs. Nevertheless, there is a very good correlation between the two chosen X-ray settings (r = 0.81). For the evaluation of bone mineral density with digital radiography in the canine femur, e.g., for follow-up studies after THR, our results indicate the importance of always choosing the same X-ray setting for one patient. Both chosen settings showed on average a good (r = 0.61) correlation with the BMD measured with DEXA. However, our correlation results differ remarkably from a previous study reporting correlation results of 0.937 between mean grey values obtained from digital radiography and BMD (DEXA) in equine bone specimens [21]. One reason for these differing results could be that in the reported study femora specimens with an overall low variability of BMD were used to calculate the correlation between DEXA and digital radiography. In contrast, our study aimed to investigate the correlation between both methods using different areas of canine femora with varying BMDs. Thus, the higher variability of BMD in our study could have been the reason for the overall lower correlation than reported by Vaccaro et al. [21] for equine bone specimens. Furthermore, it is unclear how ROI positioning was done in this specific study [21].

One factor known to influence the absorption of X-rays is the tissue surrounding the femur [22]. The surrounding tissue increases the absorption of radiation. Thus, less radiation reaches the detector and the affected pixel is given a higher grey scale value. For example, Mostafa et al. [19] measured on average 161.9–188.8 GV immediately postoperative, and 146.2–188.1 GV 4 months postoperative compared to 114.22–154.62 GV measured in our study, where femora were examined without surrounding tissues. Further studies are needed to evaluate influences of surrounding tissues. Another limitation of the study is that the influence of a total hip prosthesis on measurement results in the adjecent bone was not evaluated. Further studies are needed to investigate if the same correlations between GV (X-Ray) and BMD (DEXA) measurement like in the present study are to be found when examining canine femora with THR.

The evaluated ROIs in uncemented THR after 4 months revealed greater grey scale values than the ROIs in this study. One reason is that, due to the implanted uncemented prosthesis, the evaluated area in the study of Mostafa et al. was smaller and mainly contained compacta, while in the ROIs in this study both compacta and spongiosa were evaluated together. The other reason for differences in grey scale values is that in the study of Mostafa et al. the dogs were alive. Therefore, the radiographs were taken from the whole leg and not only from the bone. Further studies on whole legs with and without total hip implants are necessary to evaluate the influence of surrounding tissue and the metal implant (e.g., uberschwinger artefact [25]) on the measurements of GV.

## Conclusions

Results indicate that measuring absolute changes in bone mineral density in canine femoras is not possible using digital radiography due to technical limitations. Nevertheless, differences in grey scale values of the bone can be identified using digital radiography. Not all significant differences between ROIs detectable with DEXA can be displayed in the X-ray images because of the lower sensitivity of the radiographs. However, further studies are necessery to evaluate whether direct comparison of grey scale values of the periprosthetic femur in individual patients over time is possible,keeping in mind that X-rays have a lower sensitivity than DEXA. It is important that the same X-ray settings are chosen for each patient for follow-up studies.

## Additional files

Additional file 1: ml1_ml2.tif. Anterior-posterior positions ml1 and ml2. X-ray of patient no. 8 (Alsatian, 24 kg) right femur in mediolateral positions (ml1, ml2). ml1: mediolateral position, femur rotated 90° to ap1; ml2: femur rotated 90° to ap2. (TIF 1152 kb)

Additional file 2: BMD_GV_ml1_ml2.docx. Results BMD and GV. Measured bone mineral content (DEXA) and gray scale values (X-ray) for the regions of interest 1–5 (ROI1 – ROI5) in ml1 and ml2: mean value (M) ± standard deviation (SD), coefficient of variation [CV]. (DOCX 25 kb)

Additional file 3: Boxplots_ml1.tif. Box plots BMD and GV in ml1. Box plots (min to max, mean) for measured bone mineral density (BMD) in DEXA, and grey scale values (GV) in RX50 (X-ray 50 kV/6.3 mAs) and RX66 (X-ray 66 kV/8 mAs) for ROI1 – ROI5. One-Way ANOVA, * - *** statistically significant (*p* < 0.05 – *p* < 0.001). (TIF 430 kb)

Additional file 4: Boxplots_ml2.tif. Box plots BMD and GV in ml2. Box plots (min to max, mean) for measured bone mineral density (BMD) in DEXA, and grey scale values (GV) in RX50 (X-ray 50 kV/6.3 mAs) and RX66 (X-ray 66 kV/8 mAs) for ROI1 – ROI5. One-Way ANOVA, * - *** statistically significant (*p* < 0.05 – *p* < 0.001). (TIF 422 kb)

## Abbreviations

Ap: Anterior-posterior
BMD: Bone mineral density
DEXA: Dual-energy X-ray absorptiometry
E: Modulus
GV: Grey scale value
ml: Mediolateral
ROI: Region of interest
RX50: X-ray image with 50 kV and 6.3 mAs
RX66: X-ray image with 66 kV and 8.0 mAs
SD: Standard deviation
THR: Total hip replacement
WHO: World Health Organisation
