# Supplementary figures and images for: Comparative measurements of bone mineral density and bone contrast values in canine femora using dual-energy X-ray absorptiometry and conventional digital radiography
Source: BMC Vet Res. 2017 May 11;13:130. doi: 10.1186/s12917-017-1047-y (PMC5426025; doi:10.1186/s12917-017-1047-y)

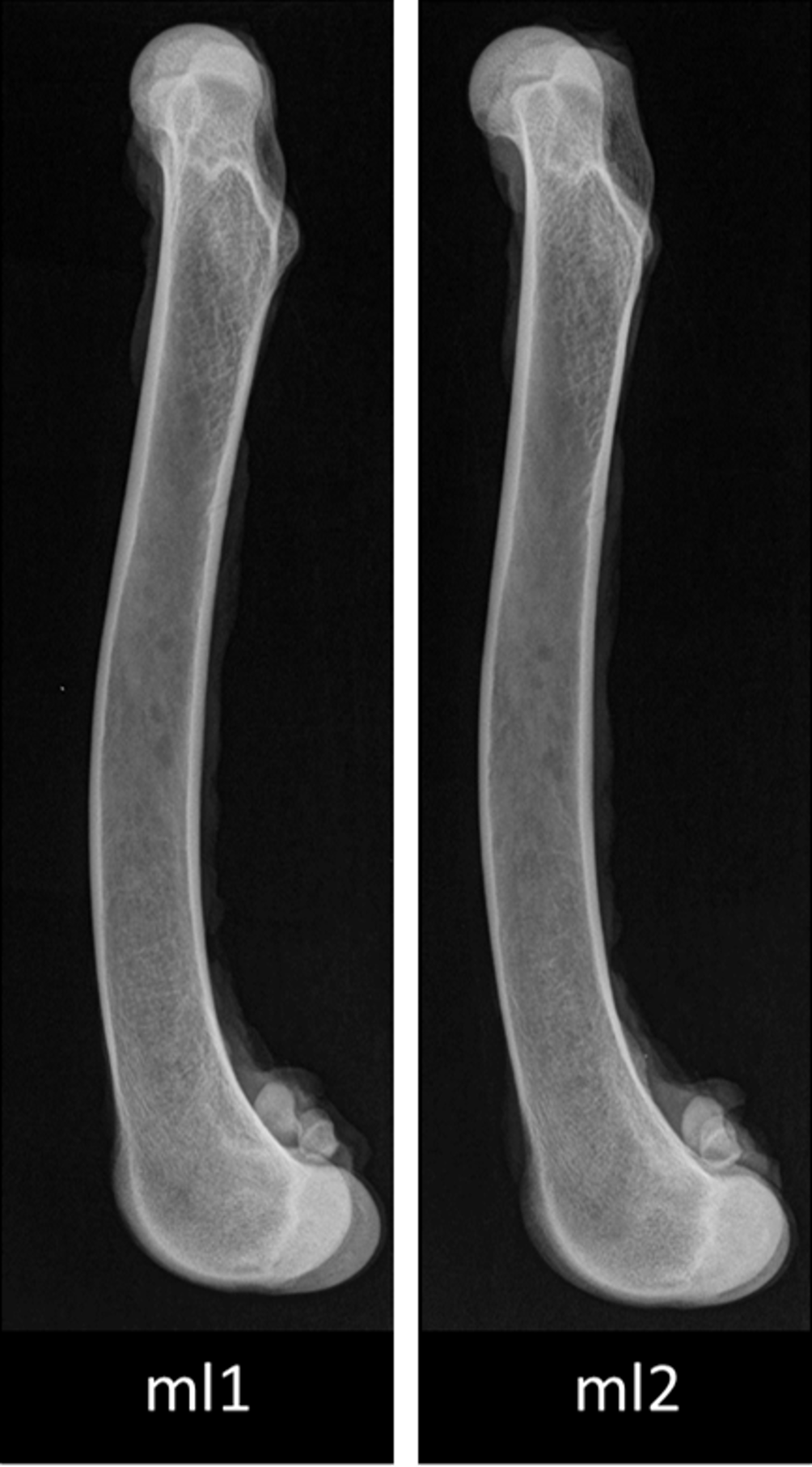

Supplement: Supplementary file 1 — ml1_ml2.tif. Anterior-posterior positions ml1 and ml2. X-ray of patient no. 8 (Alsatian, 24 kg) right femur in mediolateral positions (ml1, ml2). ml1: mediolateral position, femur rotated 90° to ap1; ml2: femur rotated 90° to ap2. (TIF 1152 kb) [file 12917_2017_1047_MOESM1_ESM.tif]

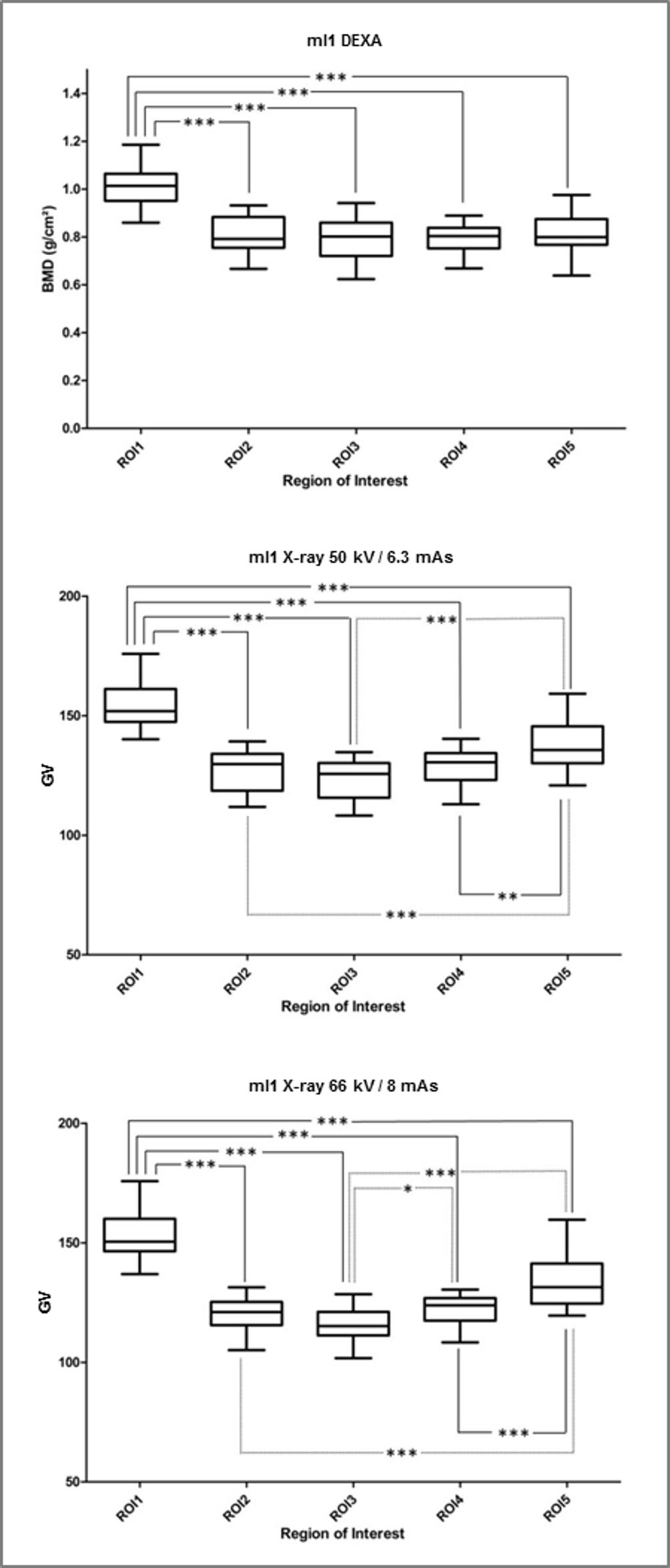

Supplement: Supplementary file 3 — Boxplots_ml1.tif. Box plots BMD and GV in ml1. Box plots (min to max, mean) for measured bone mineral density (BMD) in DEXA, and grey scale values (GV) in RX50 (X-ray 50 kV/6.3 mAs) and RX66 (X-ray 66 kV/8 mAs) for ROI1 – ROI5. One-Way ANOVA, * - *** statistically significant (p < 0.05 – p < 0.001). (TIF 430 kb) [file 12917_2017_1047_MOESM3_ESM.tif]

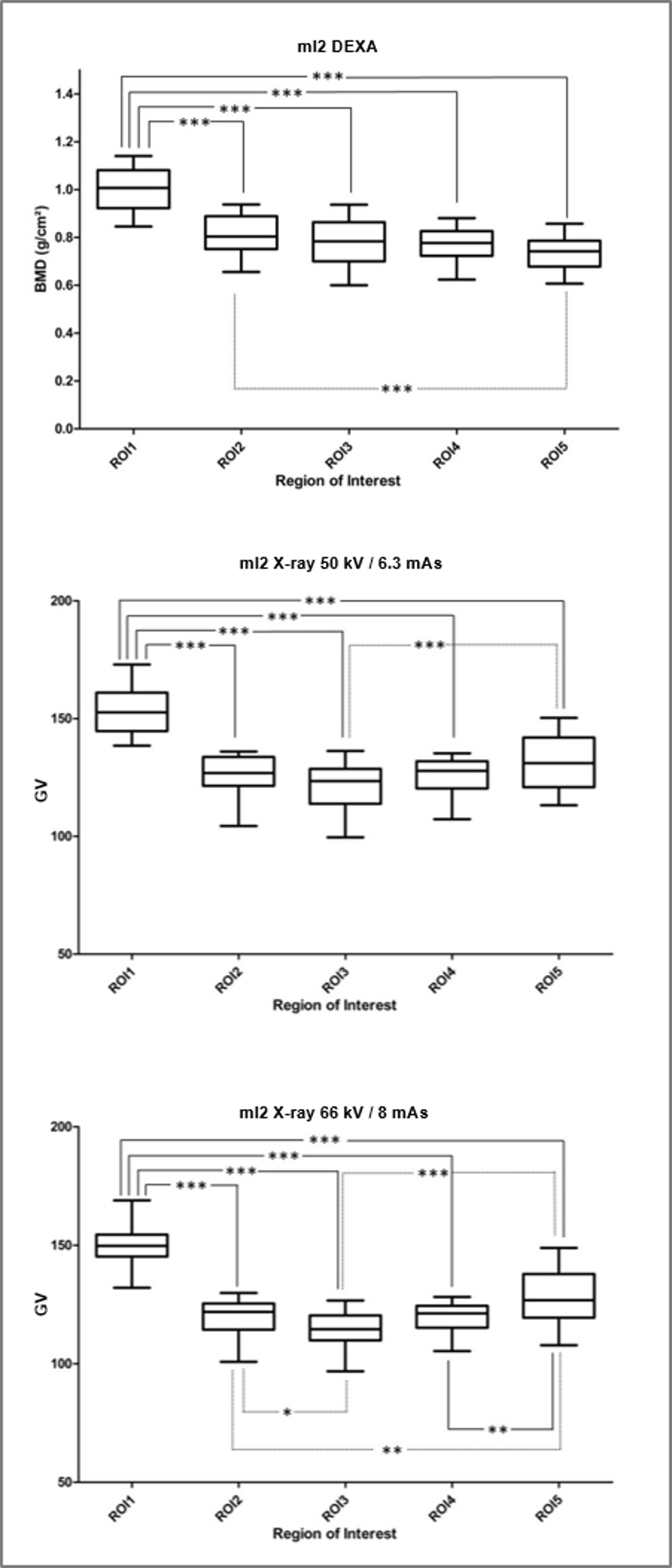

Supplement: Supplementary file 4 — Boxplots_ml2.tif. Box plots BMD and GV in ml2. Box plots (min to max, mean) for measured bone mineral density (BMD) in DEXA, and grey scale values (GV) in RX50 (X-ray 50 kV/6.3 mAs) and RX66 (X-ray 66 kV/8 mAs) for ROI1 – ROI5. One-Way ANOVA, * - *** statistically significant (p < 0.05 – p < 0.001). (TIF 422 kb) [file 12917_2017_1047_MOESM4_ESM.tif]
