# Supplementary material for: Comparative measurements of bone mineral density and bone contrast values in canine femora using dual-energy X-ray absorptiometry and conventional digital radiography
Source: BMC Vet Res. 2017 May 11;13:130. doi: 10.1186/s12917-017-1047-y (PMC5426025; doi:10.1186/s12917-017-1047-y)
Supplement: Supplementary file 2 — BMD_GV_ml1_ml2.docx. Results BMD and GV. Measured bone mineral content (DEXA) and gray scale values (X-ray) for the regions of interest 1–5 (ROI1 – ROI5) in ml1 and ml2: mean value (M) ± standard deviation (SD), coefficient of variation [CV]. (DOCX 25 kb) [file 12917_2017_1047_MOESM2_ESM.docx]

|  | **mediolateral position ml1** | | |
| --- | --- | --- | --- |
| **Region of Interest** | **DEXA**  **(g/cm²)** | **RX50**  **(gray scale value)** | **RX66**  **(gray scale value)** |
| **ROI1** | **1.02 ± 0.09 [0.08]** | **154.62 ± 9.96 [0.06]** | **152.79 ± 9.79 [0.06]** |
| **ROI2** | **0.81 ± 0.08 [0.11]** | **127.06 ± 8.64 [0.07]** | **120.05 ± 6.65 [0.05]** |
| **ROI3** | **0.80 ± 0.09 [0.11]** | **123.41 ± 8.47 [0.07]** | **115.63 ± 6.92 [0.06]** |
| **ROI4** | **0.79 ± 0.06 [0.07]** | **128.60 ±7.39 [0.06]** | **122.15 ± 6.04 [0.05]** |
| **ROI5** | **0.81 ± 0.08 [0.10]** | **137.18 ± 10.52 [0.08]** | **133.54 ± 10.66 [0.08]** |

Tab. 3 Measured bone mineral content (DEXA) and gray scale values (X-ray) for the regions of interest 1 – 5 (ROI1 – ROI5) in ml1: mean value (M) ± standard deviation (SD), coefficient of variation [CV]

|  | **mediolateral position ml2** | | |
| --- | --- | --- | --- |
| **Region of Interest** | **DEXA**  **(g/cm²)** | **RX50**  **(gray scale value)** | **RX66**  **(gray scale value)** |
| **ROI1** | **1.01 ± 0.08 [0.08]** | **153.65 ± 9.31 [0.06]** | **150.59 ± 8.22 [0.05]** |
| **ROI2** | **0.81 ± 0.08 [0.10]** | **126.59 ± 7.82 [0.06]** | **120.06 ± 6.95 [0.06]** |
| **ROI3** | **0.78 ± 0.09 [0.11]** | **121.33 ± 8.44 [0.07]** | **114.22 ±7.54 [0.06]** |
| **ROI4** | **0.78 ± 0.06 [0.08]** | **125.87 ± 7.32 [0.06]** | **119.59 ± 6.75 [0.06]** |
| **ROI5** | **0.73 ± 0.07 [0.09]** | **132.05 ± 11.02 [0.08]** | **127.90 ± 10.62 [0.08]** |

Tab. 4: Measured bone mineral content (DEXA) and gray scale values (X-ray) for the regions of interest 1 – 5 (ROI1 – ROI5) in ml2: mean value (M) ± standard deviation (SD), coefficient of variation [CV]
